# Overexpression of *CtCHS1* Increases Accumulation of Quinochalcone in Safflower

**DOI:** 10.3389/fpls.2017.01409

**Published:** 2017-08-15

**Authors:** Dandan Guo, Yingru Xue, Dongqiao Li, Beixuan He, Xinlei Jia, Xin Dong, Meili Guo

**Affiliations:** ^1^Department of Pharmacognosy, College of Pharmacy, Second Military Medical University Shanghai, China; ^2^Testing and Analysis Center, College of Pharmacy, Second Military Medical University Shanghai, China

**Keywords:** safflower, EST sequences, gene expression microarray, CHS, HSYA, transgenic safflower, quinochalcone biosynthesis

## Abstract

*Carthami flos*, the dried petal of safflower (*Carthamus tinctorius* L.) has been widely used in traditional Chinese medicine to treat cardiovascular and cerebrovascular diseases, in which quinochalcone glucosides such as hydrosafflower yellow A (HSYA), carthamin are uniquely present and have been identified as active compounds. In the present study, through sequencing of a safflower floret cDNA library and subsequent microarray analysis, we found 23 unigenes (5 *PALs*, 1 *C4Hs*, 5 *4CLs*, 6 *CHSs*, 2 *CHIs*, 2 *DFRs*, 2 *FLSs*) involved in flavonoid pathway, of which 4 were up-regulated differentially during quinochalcone glucosides accumulation with the floret developing stage. The up-regulated genes were verified by PCR methods. Considering chalcone synthase are entry enzyme in flavonoid biosynthesis, *CHS1* was focused on target gene to verify its function furtherly. Bioinformation analysis showed that *CHS1* shared 86.94% conserved residues with *CHS* from other plants. Subcellular localization showed that *CtCHS1* was localized in cytoplasm in onion epidermal cells. The transgenic safflower plant with overexpression *CtCHS1* by *Agrobacterium*-mediated pollen-tube pathway method was firstly generated. The results present that expression of *PAL2*, *PAL3*, *CHS1, CHS4, CHS6* increased and expression of *CHI1* and *CHI2* decreased in the transgenic plant floret. Meanwhile, the accumulation of quinochalcone glucosides increased by ∼20–30% and accumulation of quercetin-3-β-D-glucoside and quercetin decreased by 48 and 63% in the transgenic plant floret. These results suggested that *CtCHS1* played an important role in quinochalcone glucosides biosynthesis rather than flavonol biosynthesis. These results also demonstrated that the pollen-tube pathway method was an efficient method for gene transformation in safflower. Our study will provide a deep understanding of potential synthetic genes involved in quinochalcone biosynthetic pathway.

## Introduction

Flavonoids with a variegated structure are ubiquitous plant secondary metabolites that their physiological and ecological role is to provide relief against a large amount of biotic and abiotic stresses in the plant kingdom, such as protection against light and temperature (Winkel-Shirley, 2002), flower coloration (Tanaka and Brugliera, 2013), defense against insect infestation (El-Wakeil, 2013) and pathogen (Ortuño et al., 2006; Steinkellner et al., 2007). Meanwhile, they are recognized as abundant resources for agents that promote and maintain health (Sankari et al., 2014). Because of their significance in genetic investigations and biomedicines, flavonoid biosynthesis has attracted considerable scientific attention over the years (Giannasi, 1978; Gutha et al., 2010). However, for the extreme complexity of the secondary metabolic pathways in plants, the study on flavonoids metabolism in plants has been challenging and fascinating.

Safflower (*Carthamus tinctorius* L.) is widely cultivated as traditional herbs in China. The dried flower petal (*Carthami flos*) is a valued drug in traditional Chinese medicine and has been used clinically for a long time in China particularly for prevention and treatment of cardiovascular and cerebrovascular diseases (National Pharmacopoeia Committee, 2010). Numerous studies show that the main active compounds in safflower flower are flavonoids, such as quinochalcones (hydrosafflower yellow A, carthamin, tinctorimine, and cartorimine) and flavonols (kaempferol and its glucosides, and quercetin and its glucosides) (Tu et al., 2015). Due to natural and artificial selection for a long time, intraspecific variation from chemical components to pharmacological activity happended in safflower cultivars. For instance, ZHH0119 safflower line, which has orange-yellow petals (Y line), is a major source of quinochalcones mainly used to prevent myocardial ischemia. Whereas, the XHH007 line, which has white petals (W line), mainly contains flavonols which possessed a protective effect against cerebral ischemic damage (Li et al., 2006). To elucidate the biosynthesis mechanism of the flavonoids, several investigations on the transcriptome of safflower have been launched (Li et al., 2012). The genes encoding isochorismate synthase, cinnamate 4-hydroxylase (Sadeghi et al., 2013), oleyl-phosphatidyl-choline desaturase (*FAD2*) (Cao et al., 2013), phenylalanine ammonia-lyase and chalcone synthase (Dehghan et al., 2014), flavanone 3-hydroxylase gene (*F3H*) (Tu et al., 2016) and UDP-glycosyltransferases (*UGTs*) (Guo et al., 2016) in safflower have been cloned and characterized basically. However, due to the special nature of flavonoids, the key genes involved in flavonoids especially quinochalcones biosynthesis in safflower is hardly known. Therefore, it becomes very important to elucidate quinochalcone biosynthesis combining gene engineering method with metabolites analysis. Up to now, *Agrobacterium*-mediated pollen-tube pathway method generated the transgenic plants have been proved effective and successfully used in several species such as *Cucumis melo* L. (Hao et al., 2011; Zhang and Luan, 2016), maize (Yang et al., 2009), cotton (Wang et al., 2013). Nevertheless, no previous studies have explored transgenic saflower plant via this approach.

In the present study, in order to identify genes regulating quinochalcone glucosides biosynthesis in safflower, 23 unigenes involved in flavonoid pathways during the floret developing stage were found through sequencing of a safflower floret cDNA library. Among them, *CHS1* was focused to be an important target gene based on subsequent microarray analysis combing quantitative RT-PCR. To verify the *CHS1*’s function, we successfully generated the transgenic safflower plant with overexpression of *CtCHS1* by *Agrobacterium*-mediated pollen-tube pathway method. We found that overexpression of *CtCHS1* significantly increased the accumulation of quinochalcone glucosides in safflower, which provided a concrete evidence that *CtCHS1* is solely involved in quinochalcone glucosides biosynthesis.

## Materials and Methods

### Plant Materials

**FIGURE 1:**
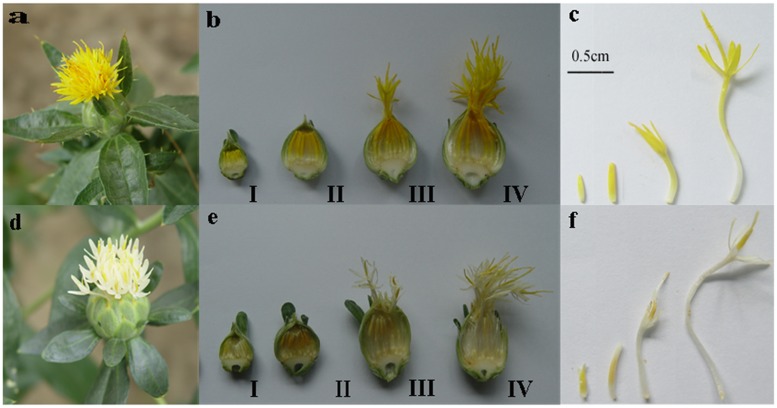
Two safflower line and their different floret developmental stage. **(a–c)** Yellow line. **(d–f)** White line. **(a,d)** Inflorescence of safflower. **(b,e)** Cross section of inflorescence from the two lines of different stages. **(c,f)** Different developmental stage of petals from two line corresponding to **(b,e)**. I: 7 days before blooming; II: 3 days before blooming; III: bloom day; IV: 3 days after blooming.

Two prominent safflower lines, ZHH0119 (Y line) and XHH007 (W line), were cultivated in the greenhouse of pharmacy college, Second Military Medical University. The temperature was set at 25°C. Circadian rhythm is 16 h/light and 8 h/darkness. Samples were picked at different floret developing stage (**Figure 1**).

### Normalized cDNA Library Construction

The early constructed normalized cDNA library in our laboratory was used in this study. RNA of equivalent different developmental stage flower samples from Y line were extracted according to manufacturer’s instructions (Invitrogen). Primary cDNA libraries were constructed with CloneMiner^TM^ cDNA Library Construction Kit (Invitrogen) and the plasmid is pDONR 222. DNA were extracted and digested by *Hin*d III and *Bam*H I. Normalized cDNA libraries were constructed by mixing DNA affinity system with primary cDNA. Normalized cDNA libraries were transformed into *Escherichia coli* cell DH10B for sequencing.

### Functional Annotation

Open reading frame (ORF) and peptide sequence were predicted by TransDecoder and ESTScan. The length distribution of unigene, ORFnn and ORFaa were proceed. Gene ontology (GO) assignments were used to classify the functions of the predicted unigenes and EST by Blast2GO. GO mapping was carried out in order to classify gene product properties into three main domains. To further study complex biological behavior of genes, ortholog assignment of unigenes were performed using Kyoto Encyclopedia of Genes and Genomes (KEGG) automatic annotation server (KAAS) with the method of bidirectional best hit (BBH). The annotation can help us further understand the role of genes in signal pathway.

### Gene Chip

RNA of equivalent different developmental stage flower samples from two safflower lines were isolated using trizol and purified. Hybridization of the RNA was performed using the gene expression hybridization kit (Agilent). 8 × 15K (Agilent) *in situ* synthesis of gene chip was used to detect gene expression. Then the microarrays were scanned with the Microarray Scanner (Agilent). gProcessed Signal were the value of transcript abundancy. The expression analysis of unigenes in two lines were performed by DESeq (V1.14.0) (Gentleman et al., 2004). Differential genes were screened out with appropriate fold change absolutely (FCA) calculated and p-value. Hot map for expression signal intensity and hierarchical clustering (HCL) were performed by MeV (Eisen et al., 1998).

### Semi-Quantitative RT-PCR/Quantitative RT-PCR Analysis

Total RNA was extracted by trizol (TransGen Biotech, Beijing, China) according to manufacturer’s protocol. The first-strand cDNA were synthesized with 500 ng RNA according to cDNA Synthesis SuperMix (TransGen Biotech, Beijing, China). The specific primers of genes in flavonoid biosynthesis were designed using Beacon Designer 8 (Supplementary Table S1). The quantitative RT-PCR was performed by use of the SYBR Green Realtime Master Mix kit (Transgene, China), and carried out with ABI 7500 detection system (ABI, United States). PCR conditions is the same as those described previously (Guo et al., 2016). All quantitative real-time PCR amplifications were carried out with three independent biological replicates. The specificity of amplification was assessed by dissociation curve analysis. And the relative expression level of genes was determined using the 2^-ΔΔCt^ method. Semi-quantitative RT-PCR amplification of RNA was performed as previously reported without curve stage. The *Ct60S* gene (KJ634810) was treated as reference gene.

### Clone and Bioinformatic Analysis of *CtCHS1*

cDNA library was built with SMARTer^TM^ RACE cDNA Amplification Kit (Clontech, United States). Gene-specific primers (GSPs) were designed (*CtCHS1*-GSP1-5′:CGCTCGTGTCCTCGTGGTTTGCTC; *CtCHS1*-GSP1-3′:CAACGGAGAAAACGCCTGC). The sequences of the 3′- and 5′-RACE products were produced by Advantage 2 PCR Kit (Clontech, United States) and sequenced. To obtain the full-length sequence of *CtCHS1*, primer was designed by Oligo 7 (*CtCHS1*-full-length-5′:ATGGCATCCTTAACCGATATTG; *CtCHS1*-full-length-3′:TTAAGCGGCAATGGGGGTGG). PCR was performed using KOD-Plus-Neo polymerase mix system (Toyobo, Japan) when the condition is: 30 cycles of 10 s denaturation at 94°C, 30 s annealing at 58°C, and 60 s amplification at 72°C. The PCR products were purified (QIAquick^®^ Gel Extraction Kit, Qiagen, Germany) and cloned into the PMD-19T vector (Takara, Japan). Full length of *CtCHS1* was sequenced. The obtained ORF sequence and deduced protein were analyzed using websites^1^,^2^. Multiple sequence alignment was performed by DNAMAN and phylogenic tree was built by using ClustalX version 2.0 (Larkin et al., 2007) and MEGA (Tamura et al., 2011).

### Subcellular Localization

The entire coding sequence (CDS) of *CtCHS1*was amplified with primers (*CHS1*-F:GAGCTTTCGCGGATCCGCCACCATGGCATCCTTAACCGATATTG and *CHS1*-R:CATGGTGGCAAGCTTAGGGCCGGGATTCTCCTCCACGTCACCGCATGTTAGAAG). The tool vector pCAMBIA1380-GFP (green fluorescent protein) was digested by incorporated *Bam*HI-HF and *Spe*I-HF sites. The PCR product was cloned into the vector pCAMBIA1380-GFP to generate pCAMBIA1380-*CtCHS1*-GFP fusion protein construct driven by the cauliflower mosaic virus (CaMV) *35S* promoter. The resulting plasmids were confirmed by sequencing and further transformed into the *Escherichia coli* DH5a. The culture process of onion epidermal layers was performed according to previous methods (Tu et al., 2016). A localization assay was carried out as described by Mare et al. (Mare et al., 2004). The localization of the fusion protein was observed by using of a confocal microscope (Leica TCS SP5). GFP fluorescence, the bright field image was shot simultaneously and merged together.

### Genetic Manipulation

The ORF of the *CHS1* gene was cloned with primers above. CaMV*35S* promoter is the most commonly promoter to drive expression of transgenes in plants (Odell et al., 1985). Empty vector PMT39 (**Supplementary Figure S1**) was digested by *Hin*d III and *Bam*H I and linearized recombinant vector was constructed under the control of the *35S* promoter with seamless cloning reaction mix according to the manufacturer’s instructions (Takara), which was then transformed into *Agrobacterium* LBA4404 by electroporation. Transformant cells were grown in LB medium with 50 mg/L kanamycin and 100 mg/L streptomycin. The primer (F:ATCTCTCTCGAGCTTTCGCGG; R:CGTCGCCGTCCAGCTCGACCAG) was designed to screen positive transformants.

### Genetic Transformation of *CtCHS1* in Safflower

A single colony was grown in 2 ml LB medium containing 50 mg/L kanamycin and 100 mg/L streptomycin with shaking at 30°C and confirmed by PCR. Then the culture was inoculated in 50 ml fresh LB medium containing kanamycin and streptomycin. It was grown at 30°C with 1 μM acetosyringone when optical density of the culture reached to ^∼^0.6–0.8 at 600 nm. The resulting cells were harvested by centrifugation at 5000 × *g* for 10 min and resuspended in 5% sucrose solution while adding 0.02% silwet-L 77. The solution harboring the PMT39-*CHS1* vector is used right now after it is ready for genetic transformation of wild-type Y safflower line (T0) grown in greenhouses. After the flowers were pollinated and before the upper part of the stigma was closed, the flowers were then immediately injected with a solution containing plasmid DNA, using a microsyringe with the needle kept in a vertical position. When finished, the floret is placed at dark condition for 12 h by covering them with bags. Seeds of T0 were cultivated and T1 generation with over-expression *CtCHS1* were obtained.

### UPLC/Q-TOF/MS Detection in Transgenic Safflower

Samples were dried to constant weight at 50°C and ground into powder immediately. Subsequently, an aliquot of 5 mg samples was soaked overnight with 60% methanol under sealed conditions and then extracted for 40 min sonication (Guo et al., 2016). Then, the supernatant was analyzed. The metabolites were identified using an ultra high-performance liquid chromatography (UPLC) system (Agilent 1290 Infinity UPLC; Agilent Technologies, Waldbronn, Germany) fitted with the Agilent 6538 UHD Accurate-Mass Q-TOF LC/MS (Agilent Technologies, Santa Clara, CA, United States) equipped with an ESI (electrospray ionization) interface. Waters XSELECT HSS T3 C18 column (100 × 2.1 mm, 2.5 μm). Mobile phase A is water with 0.1% formic acid and mobile phase B is acetonitrile with 0.1% formic acid. Flow rate is 0.4 ml/min, column temperature was held at 40°C. The gradient elution, mass spectrometer and positive ion mode used for the quantification were performed as described previously. The 11 standard compounds were confirmed, namely: D-phenylalanine [m/z 165.0790], rutin [m/z 610.1534], quercetin 3-β-D-glucoside [m/z 464.0955], kaempferol-3-*O*-β-D-glucoside [m/z 448.1006], luteolin [m/z 286.0477], apigenin [m/z 270.0528], naringenin [m/z 272.0685], quercetin [m/z 302.0427] and kaempferol [m/z 286.0477] purchased from Sigma–Aldrich (St. Louis, MO, United States), HSYA [m/z 612.1690] and carthamin [m/z 910.2168] extracted in our lab. Metabolites data were proceed with MassHunter quantitative analysis software (Agilent).

## Results

### Sequence Assembly

Normalized cDNA library was built successfully with colony-forming unit (CFU) of 2.77 × 10^5^, recombination rate of >95%, and average insertion fragment length of >1.2 kb. 5′ end sequencing of clones from library resulted in a total of 32,299 EST sequences. 804 empty sequences by Cross_Match, 100 sequences shorter than 100 bp, 144 embedded sequences and 136 longer than 20bp poly-A were removed. Assessment of these EST sequences revealed that a total of 31,130 are “good quality” sequences. In order to assess EST redundancy, masked EST sequences were pair-wise compared and grouped into clusters, based on sequence similarity (Alagna et al., 2009). Therefore, the obtained clusters are ESTs which are most likely products of the same gene. Each cluster was then assembled into one or more tentative consensus sequences (TCs), which were derived from multiple EST alignments. ESTs did not fit the match criteria to be clustered/assembled with any other EST, were defined as singletons. The combination of TCs are referred to as contigs. Assembling these ESTs by CAP3 resulted in the identification of 7,737 unigenes (4,016 contigs and 3,721 singletons). The length distribution of unigenes, ORFnn and ORFaa were present (Supplementary Table S2).

### Functional Annotation

**FIGURE 2:**
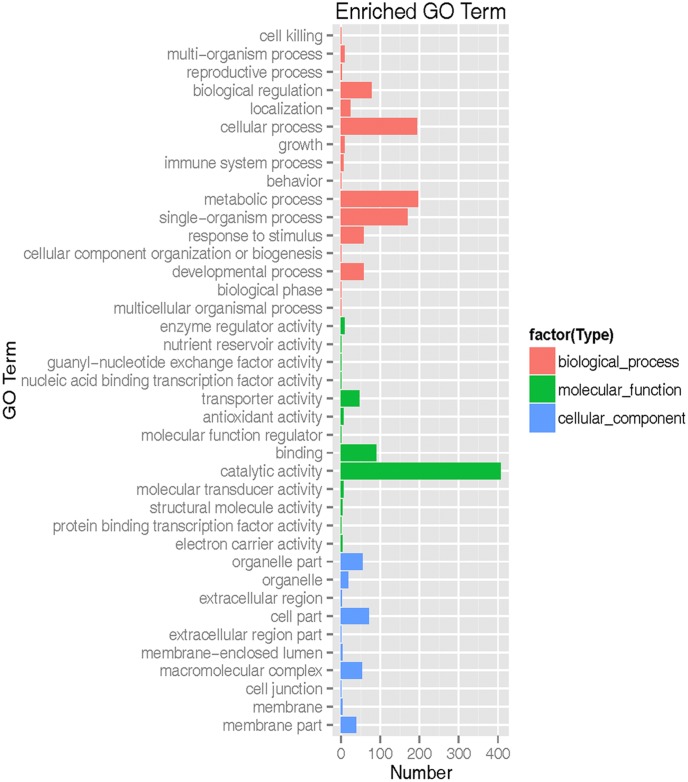
Gene ontology (GO) functional classification of safflower. Red frame is biological process. Green frame is molecular function. Blue frame is cellular component.

**FIGURE 3:**
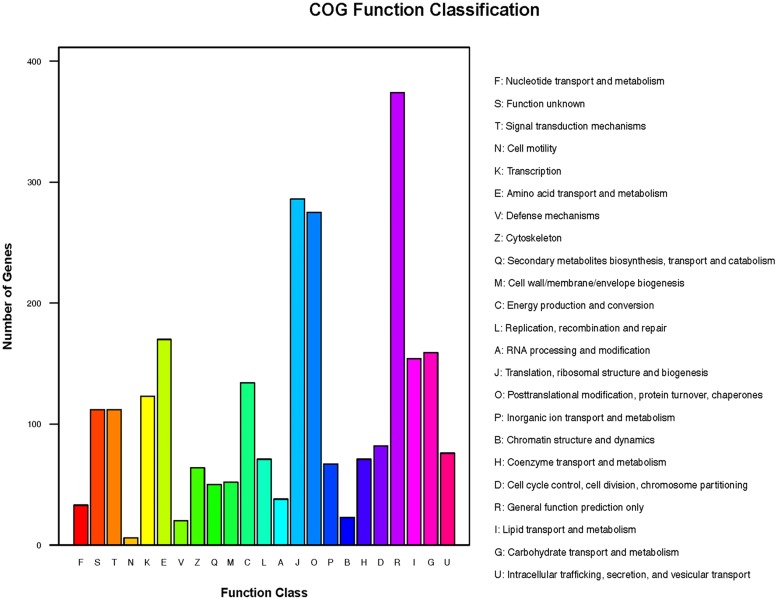
Cluster of orthologous gene (COG) functional annotations of safflower. A total of 23 function classes were described.

The transcripts were annotated using NCBI BLAST, GO, Cluster of orthologous gene (COG) and KEGG. Based on shared high homology with sequences in the public databases, blastx and blastn analysis revealed 6,755 unigenes with annotation and 982 without annotation in Nr database and 4,240 unigenes with annotation and 3,497 without annotation in Nt database (*E*-value < 1 × 10^-5^). GO^3^ classification revealed 2,237 unigenes took part in molecular function, cellular component and biological process (**Figure 2** and Supplementary Table S3). To further predict the function of unigenes, they were classified into different protein families based on COG protein databases. Overall 5,429 annotated unigenes were assigned to the appropriate COG clusters phylogenetically. The COG annotated proteins were functionally distributed into 23 protein families (**Figure 3** and Supplementary Table S4), of which the cluster of “general function prediction” possessed the largest group (374 unigenes), followed by “translation, ribosomal structure and biogenesis” (286 unigenes), “posttranslational modification, protein turnover, chaperones” (275 unigenes), “amino acid transport and metabolism” (170 unigenes) and “carbohydrate transport and metabolism” (159 unigenes). KEGG pathway database intend to systematic analysis of functions of gene products and inner-cell metabolic pathways (Rama Reddy et al., 2015). The mapping of KEGG functional pathway categories indicated that a total of 3011 annotated unigenes assigned 304 pathways (Supplementary Table S5). Among them, a total of 23 unigenes were involved in flavonoid biosynthesis, which respectively contained unigenes for 5 phenylalanine ammonia-lyase unigenes (*PAL*), 1 cinnamate-4-hydroxylase unigenes (*C4H*), 5 4-coumarate: CoA ligase unigenes (*4CL*), 6 chalcone synthase unigenes (*CHS*), 2 chalcone isomerase unigenes (*CHI*), 2 dihydroflavonol 4-reductase unigenes (*DFR*), 2 flavonol synthase (*FLS*).

### Gene Expression Microarray Analysis

**FIGURE 4:**
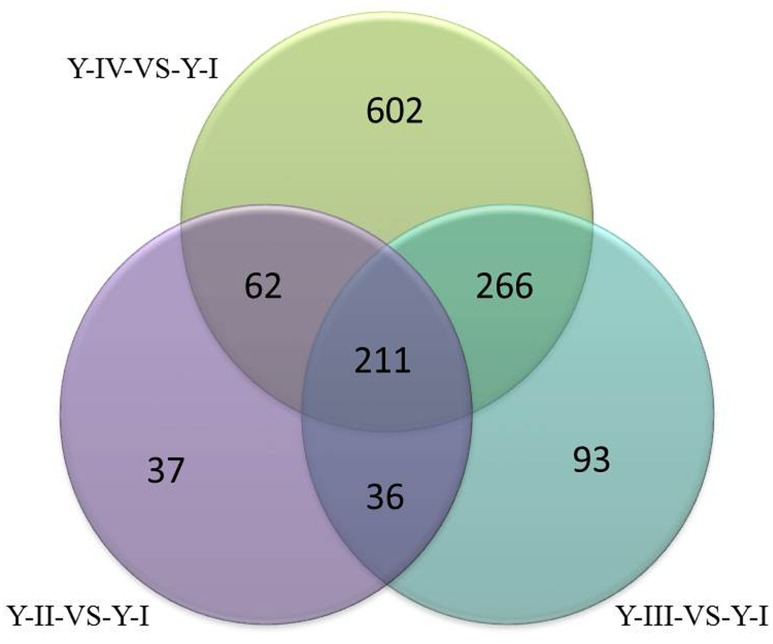
Venn diagram of up-regulated genes (*p* ≤ 0.05) at different developmental stage of flower in Y line.

**FIGURE 5:**
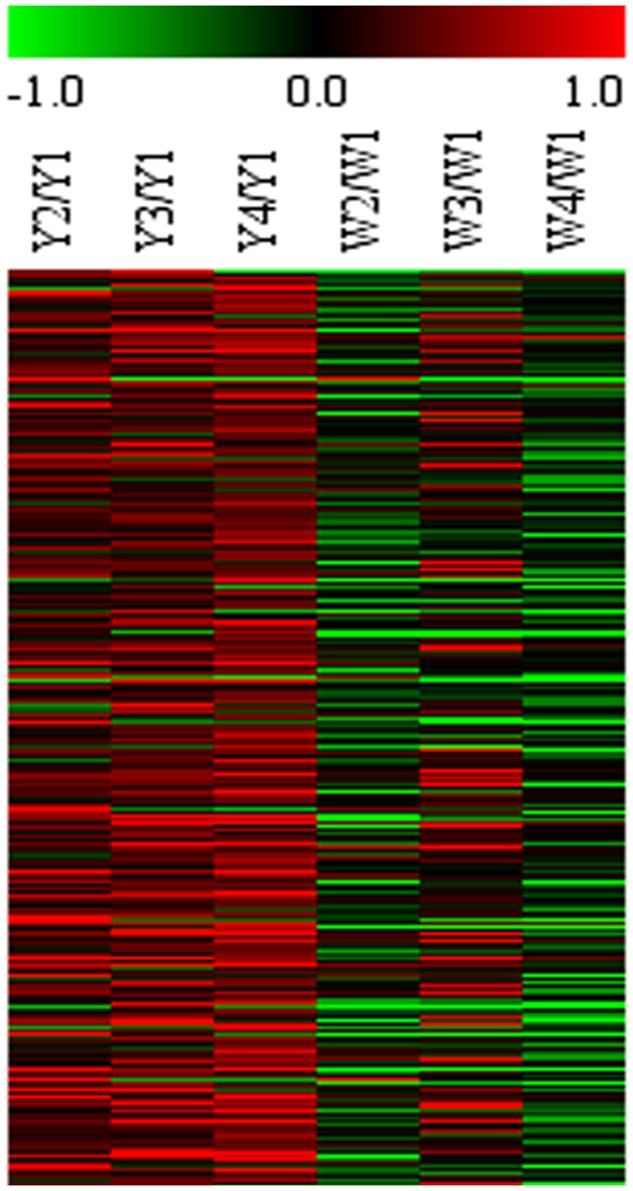
Microarray expression abundance of differential genes at different flower stages in Y and W line. HCL cluster of genes was performed. The color key was set from –1 to +1. The data were retrieved from the safflower microarray data set (unpublished).

To select differential gene, gene expression was analyzed to set FCA ≥ 2 and the *p*-value ≤ 0.05 in different sample. In Y line, comparing to Y-I stage, 346 unigenes were up-regulated and 58 were down-regulated in Y-II stage, 606 unigenes were up-regulated and 260 unigenes were down-regulated in Y-III stage and 1141 unigenes were up-regulated and 483 unigenes were down-regulated in Y-IV stage. A total of 1,307 unigenes were up-regulated with union (**Figure 4**). GO terms analysis revealed that the largest cluster is molecular function which 279 unigenes took part in, while 190 unigenes were involved in biological process and 74 unigenes in cellular component. KEGG results showed that 361 pathway with annotation by union set, in which, the starch and sucrose metabolism (ko00500) was the largest pathway cluster (31, 7.83%), in addition, phenylalanine metabolism pathway (ko00360) contained 17 annotated unigenes (4.29%) and monoterpenoid biosynthesis (ko00902) included 3 annotated unigenes (0.76%). In W line, comparing to W-I stage, 1960 unigenes were identified as up-regulated genes with a union of W-II/W-I, W-III/W-I and W-IV/W-I up-regulated unigenes. Comparing to the whole of up-regulated unigenes in W line (*p* ≤ 0.25), 266 up-regulated unigenes with specificity existed in Y line (**Figure 5**). Among them, 4 flavonoid-related specific genes were identified, included *PAL3*, *CHS1*, *FLS1* and *FLS2*.

### Quantitative Real-time PCR/Semi-Quantitative RT-PCR Analysis

**FIGURE 6:**
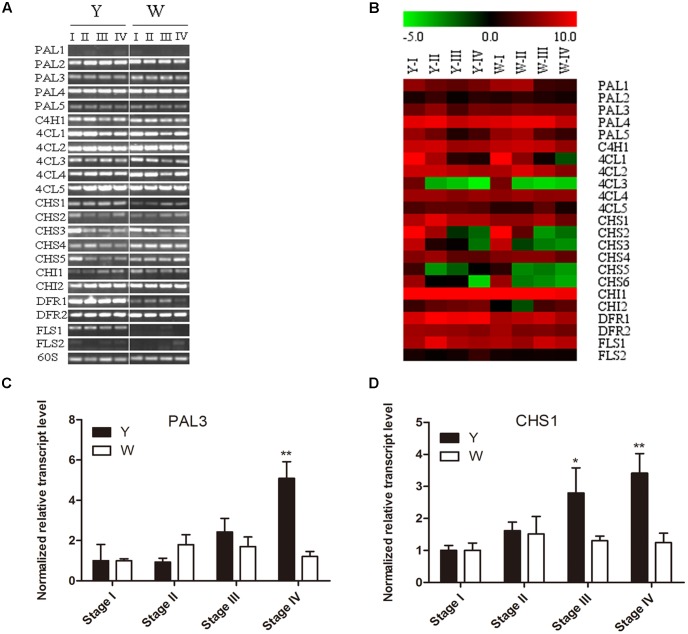
Expression pattern of flavonoid-related genes at flower different developmental stage of two lines. **(A)** Detected by semi-quantitative RT-PCR. **(B)** Signal values by gene chip, W1 and Y1 as control group. The color key was set from –5 to +10. **(C)** qPCR of PAL3. **(D)** qPCR ofCHS1.

To further confirm the differential genes between Y line and W line, Semi-quantitative RT-PCR was performed with 1.5% agarose gel. The expression pattern of 23 flavonoid-related genes was examined at flower different developmental stage of two lines in **Figure 6A**. The result shows that *PAL2*, *PAL4*, *4CL1*, *4CL2*, and *4CL4*, *4CL5*, *CHI1*, and *DFR2* are high expressed while the expression of *PAL1* and *FLS2* are weak in both two lines. However, *CHS1*, *DFR1* and *FLS1* have higher transcript level in Y line comparing with W line macroscopically. To confirm the difference of *PAL3* in two lines, qPCR performed showed that *PAL3* only has up-regulation trend at latest stage of flower in Y line, which is not as differential gene (**Figure 6C**). Combining with gene chip results (**Figure 6B**), only three important differential genes, *CHS1*, *DFR1*, and *FLS1*, were screened out. Chalcone synthase is an entry enzyme in flavonoid biosynthesis pathway, which not only participate in quinochalcone biosynthesis but also flavonol biosynthesis. In safflower two lines, quinochalcone is the main metabolites in Y line and flavonol is the principal compounds in W line. As a bridge of metabolites tributary, *CHS1* was focused on an important target gene to verify its function.

### Molecular Cloning and Characterization of *CtCHS1*

**FIGURE 7:**
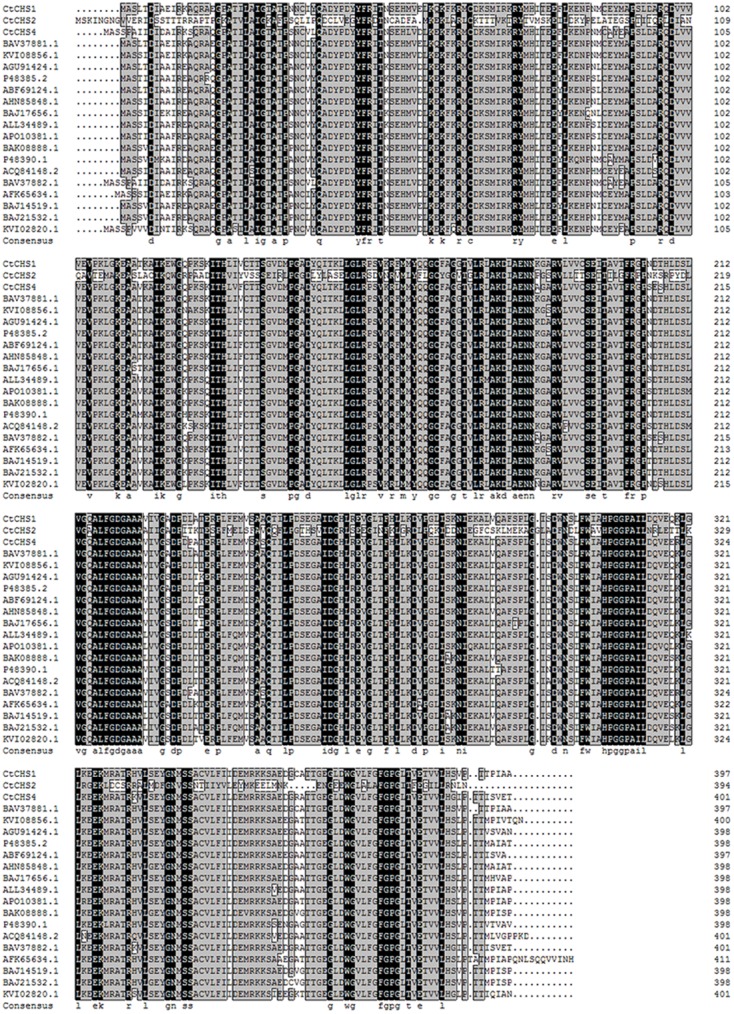
Alignment of deduced amino acid sequences of plant CHS proteins. Identical residues are highlighted on a black background, and similar residues are highlighted on a gray background. The GenBank accession numbers are as follows: BAV37881.1 and BAV37882.1 from *Carthamus tinctorius*, KVI08856.1 and KVI02820.1 from *Cynara cardunculusvar. scolymus*, AFK65634.1 from *Silybum marianum*. AGU91424.1 from *Chrysanthemum boreale*, P48385.2 from *Callistephus chinensis*, ABF69124.1 from *Chrysanthemum x morifolium*, AHN85848.1 from *Eschenbachia blinii*, BAJ17656.1 from *Gynura bicolor*, ALL34489.1 from *Helianthus annuus*, BAJ14519.1, BAJ21532.1 and BAK08888.1 from *Dahlia pinnata*, APO10381.1 from *Echinacea pallida*, P48390.1 from *Gerbera hybrida*, ACQ84148.2 from *Ageratina adenophora*.

**FIGURE 8:**
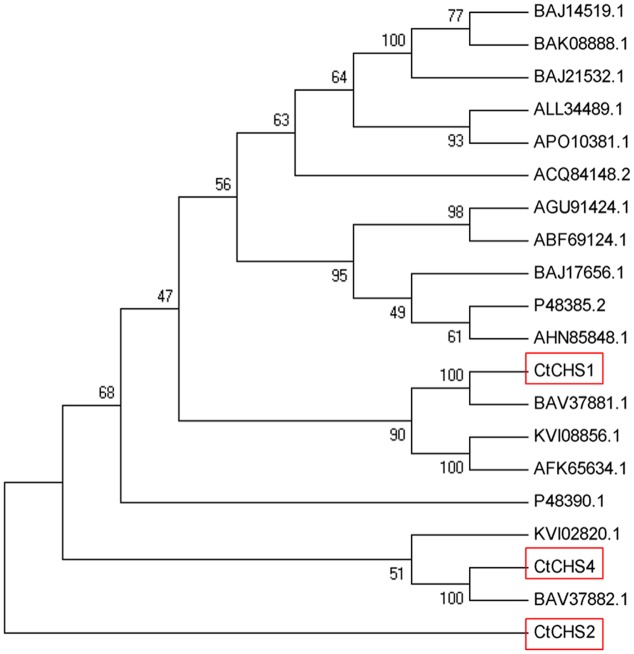
Unrooted phylogram comparison of the amino acid sequences of *Ct*CHS1 and other functionally characterized CHS proteins. The sequences used are the same as in figure. The phylogenetic tree was constructed by MEGA, after alignment using ClustalX software. Node support was estimated using neighbor-joining bootstrap analysis (1,000 bootstrap replicates).

The full-length nucleotide sequence of *CtCHS1* was predicted to be 1,487 bp (GenBank accession no. KY471385). In addition, to verify further the differential expression of *CtCHS1*, qPCR of full-length of *CtCHS1* was carried out which confirmed *CtCHS1* have higher transcript level at the late stage in Y line than W line (**Figure 6D**). *CtCHS1* encodes a predicted protein of 398 amino acids, with a calculated pI of 6.11 and a molecular mass of 43.4 kD. BLASTX analysis of the *CtCHS1* sequence against the GenBank database showed that the deduced amino acid sequence of *CtCHS1* showed 86.94% identity to other known CHS proteins from different plant species, such as KVI08856.1from *Cynara cardunculus var. scolymus* and AFK65634.1 from *Silybum marianum* (**Figure 7**). Phylogenetic analysis (**Figure 8**) showed that *CtCHS1* has 100% similarity with BAV37881.1 from safflower. In addition, *CtCHS2* and *CtCHS4* from safflower are less than 72% identity to *CtCHS1*. *CtCHS2* is less closely related to CHS protein from other species at the amino acid level. This suggested that the 3 CHS proteins may possess different biological functions in safflower.

### Subcellular Localization of *CtCHS1*

**FIGURE 9:**
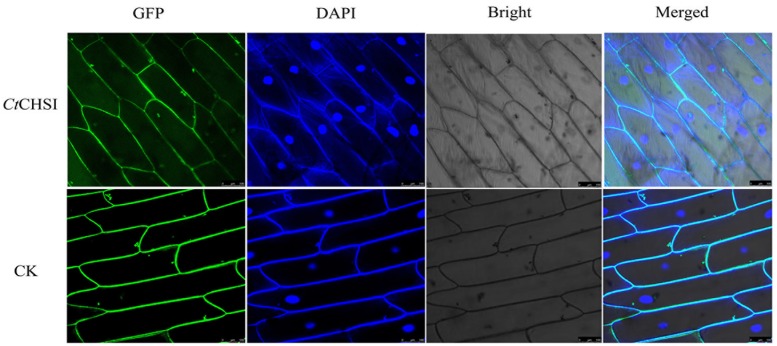
Subcellular localization of the *Ct*CHS1-GFP fusion protein in onion epidermal cell. GFP fluorescence, bright field and an overlay of bright and GFP fluorescence illumination (merged) are shown.

It is important to elucidate the functional roles in plant cells by analyzing the subcellular localization of proteins. The probable subcellular localization of *CtCHS1* was computationally analyzed by using the WoLF PSORT program^4^ (Horton et al., 2007), and the results indicated that *CtCHS1* may be positioned at the cytoplasm. To examine the localization of the *CtCHS1* protein, a *CtCHS1*-GFP fusion construct was introduced into onion epidermal cells. The results displayed that *CtCHS1*-GFP protein was detected mainly in the cytoplasm and cytomembrane in **Figure 9**, while the control (transformation of GFP construct) was observed in the cytomembrane. The results indicated that *CtCHS1* may be localized in cytoplasm.

### Transcriptional Expression of Associated Genes in Transgenic Safflower

**FIGURE 10:**
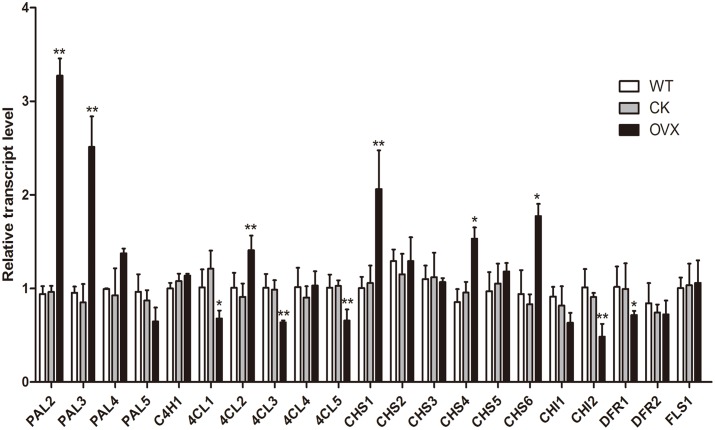
Transcript levels in wild-type and *Ct*CHS1 over-expression safflower as determined by qRT-PCR. WT, wild-type safflower line. CK, safflower line with empty-vector control. OVX, *Ct*CHS1 Over-expression safflower line. CK group VS WT group, OVX group VS CK. ^∗^*p* ≤ 0.05, ^∗∗^*p* ≤ 0.01.

The *CtCHS1* gene driven by the 35S promoter was introduced into safflower by *Agrobacterium* tumefaciens mediated transformation. Eight out of 31 independent positive transgenic lines were screened out by PCR. Transcript levels of genes involved in flavonoid biosynthesis pathway were further analyzed by qRT-PCR in wild-type and transgenic plants. Comparing with empty-vector plant group, *CtCHS1* transcript level raises significantly with about 0.95 fold in transgenic plants. Meanwhile, as members of chalcone protein family of safflower, transcript level of *CHS4* and *CHS6* was also up-regulated by 0.60 and1.14 fold. Transcript level of *CHS2* and *CHS5* also had an upward trend, though it did not reached a significant level. Transcript level of up-stream pathway genes, *PAL2* and *PAL3* raised significantly with about 2.40 and 1.95 fold, respectively (**Figure 10**). While *4CL1*, *4CL3*, *4CL5* declined significantly with about 0.44, 0.35, and 0.36 fold. In addition, *PAL1*, *FLS2* with low transcript signal in wild-type plant would not be discussed here. On the contrary, down-stream pathway genes *CHI1*, *CHI2* were suppressed by 0.22, 0.47 fold. This indicated that over-expression of *CtCHS1* in Y line had promoted transcript of its family (*CHS4* and *CHS6*) and up-stream genes (*PAL2* and *PAL3*) and restrained expression of down-stream genes. From these results, we thought that over-expression of *CtCHS1* would result in over-expression of most of its family member, *PAL2* and *PAL3*, participated in quinochalcone glucosides biosynthesis as the co-regulators of *CtCHS1* and inhibit transcript expression of *CHI1* and *CHI2*, giving some clues for *CHI* not participating in quinochalcone glucosides biosynthesis in safflower.

### Profiling of Flavonoids Accumulation in Transgenic Safflower

**FIGURE 11:**
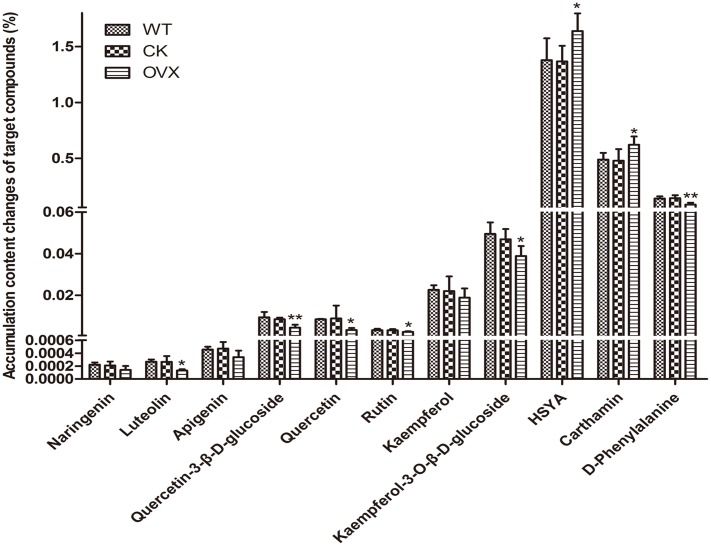
Levels of target flavonoid compounds in wild-type and transgenic safflower determined by UPLC-MS analysis. The data represent the content corresponding to each compound by that of the standard curve method. Results are presented as means mean ± SD from biological triplicates. CK group VS WT group, OVX group VS CK. ^∗^*p* ≤ 0.05, ^∗∗^*p* ≤ 0.01.

**FIGURE 12:**
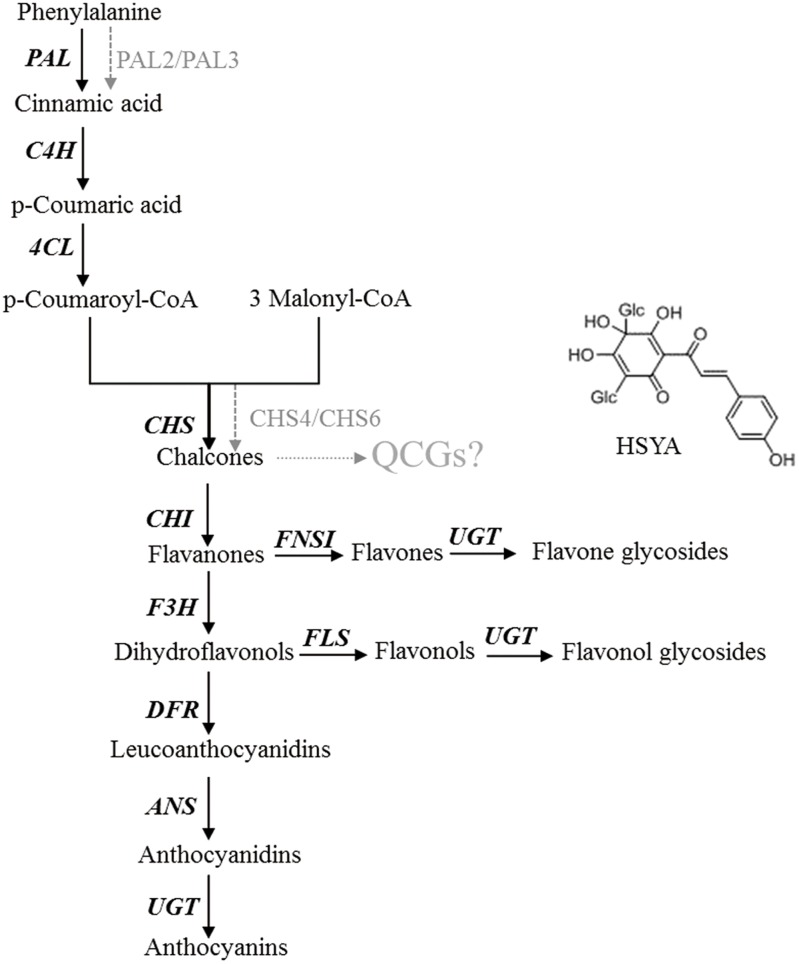
Schematic for the putative biosynthetic pathways of quinochalcone C-glucosides in safflower

To determine whether overexpression of this gene could modulate HSYA biosynthesis, we introduced *CtCHS1* into Y safflower line. The transgenic plant line did not display obvious phenotypical differences compared with wild-type line. To further investigate the impact of *CtCHS1* overexpression on flavonoid biosynthesis, levels of flavonoid-skeleton secondary metabolites were measured by ultra-high-performance liquid chromatography coupled to electrospray ionization quadrupole time-of-flight mass spectrometry (UPLC-ESI-QTOF-MS) in **Supplementary Figure S2**. The quantitative of compounds were analyzed by using standard curve method. The average contents of three biological replicates were presented. Over-expression of *CtCHS1* have brought that all the flavonols detected were down-regulated. The greatest effects were detected in flowers, where levels of quercetin and its glucoside (**Figure 11**) as well as luteolin were reduced by 47–63% in the transgenic lines. Kaempferol and its glucosides were down-regulated by 14.41% (kaempferol) and 17.06% (kaempferol-3-*O*-β-D-glucoside). In contrast, quinochalcone C-glucosides such as HSYA and carthamin, were increased apparently by 19.83 and 29.48% in *CtCHS1* over-expression line. Surely, as the precursor of flavoniods, D-phenylalanine is consumed faster 39.51% resulted from over-expression of *CtCHS1*. This is consistent with the transcript level change *CtCHS1* over-expression aroused (**Figure 10**). The result reveals that *CtCHS1* opens a tributary to quinochalcone biosynthetic pathway and block off *CtCHIs* that flow to downstream pathways to synthesize various flavonols aglycones and glycosides suggesting that *CtCHS1* solely take part in quinochalcone biosynthetic pathway rather than flavonol (**Figure 12**).

## Discussion

Quinochalcone glucosides such as HSYA, carthamin are uniquely present and have been identified as active compounds in safflower. However, little is known about its biosynthesis. With the boom of transcriptome sequencing, high-throughput data and plant transgenic technology, it has become possible to verify the complex biosynthesis process of specialized metabolites in safflower.

CHS is the branch-point toward various flavonoids, which converts 1 *p*-coumaroyl-CoA and 3 malonyl-CoA into naringenin chalcone in previous studies. The detailed steps toward unique quinochalcone have not yet been proven. To elucidate the key genes of quinochalcone glucosides biosynthesis in safflower, *CHS1* was focused to be a key gene taking part in quinochalcone glucosides biosynthesis in safflower through sequencing of a safflower floret cDNA library, analysis of subsequent microarray and overexpression of *CtCHS1*.

We cloned full length and ORF of *CHS1* at the level of transcript and found that it shared 86.94% conserved residues with CHS from other plants. *CtCHS1* was localized in cytoplasm which offering some clues for its functional characterization in the biological processes of plant. Junichi Shinozaki (Shinozaki et al., 2016) reported that 3 *CHSs* has a typical chalcone synthase activities *in vitro* in safflower. Nevertheless, the functions of *CHSs* in flavonid pathway were still largely unknown.

To verify the *CHS1*’s function in safflower, we firstly generated the transgenic safflower plant by *Agrobacterium*-mediated pollen-tube pathway method. We found that flavonoid gene family have different reactions responding to over-expression of *CtCHS1*.*PAL2*, *PAL3*, *4CL2*, *CHS4* and *CHS6* are positive co-regulation factor with *CHS1*, while *4CL1*, *4CL3*, *4CL5*, *CHI2* and *DFR1* are repressed responding to over-expression of *CtCHS1*. Meanwhile, over-expression of *CtCHS1* has brought that quinochalcone glucosides (HSYA and carthamin) increasing apparently in the transgenic lines and the down-regulated flavonols aglycone and glucosides. This indicated that *CtCHS1* starts a unique flux to modulate specially quinochalcone glucosides biosynthetic pathway.

## Conclusion

This study demonstrates that overexpression of *CtCHS1* increases the quinochalcone glucosides accumulation and decreases flavonols aglycones and glycosides contents in safflower for the first time. *CtCHS1* takes part in quinochalcone biosynthetic pathway rather than flavonol. Surely, the numerous functional enzymes flowing to complex quinochalcone glucosides remain to be explored, including UDP-glycosyltransferase, transcription factors, cytochrome P450, etc. Our findings are instructive for understanding the characterization of the CHS genes aimed at elucidating the molecular mechanisms involved in quinochalcone biosynthesis. We will deeply study the relevant genes of regulating quinochalcone synthetic pathway by bioengineering methods to clarify chalcones synthesis in safflower.

## Author Contributions

YX and DL clone the gene, BH and XJ contributed to the collection of plant sample and DNA and RNA extraction. MG and DG carried out the annotation of unigenes and gene chip data and wrote the manuscript. DG was responsible for qRT-PCR and bioinformatics analysis and metabolites data. XD analyzed the metabolites. MG designed the experiment and initiated the project. All the authors have read the final manuscript and approved the submission.

## Conflict of Interest Statement

The authors declare that the research was conducted in the absence of any commercial or financial relationships that could be construed as a potential conflict of interest.

## Footnotes

1 www.ncbi.nlm.nih.gov

2 www.expasy.org

3 http://amigo.geneontology.org

4 http://www.genscript.com/psort/wolf_psort.html

## Supplementary Material

FIGURE S1 Plasmid map.

FIGURE S2 Representative total ion chromatograms (TICs) in ESI negative ion mode based on UPLC-Q-TOFMS from WT, CK and OVX in safflower. **(B)** EICs of each 11 metabolites from WT, CK and OVX in safflower.
