# Supplementary figures and images for: Overexpression of CtCHS1 Increases Accumulation of Quinochalcone in Safflower
Source: Front Plant Sci. 2017 Aug 15;8:1409. doi: 10.3389/fpls.2017.01409 (PMC5559696; doi:10.3389/fpls.2017.01409)

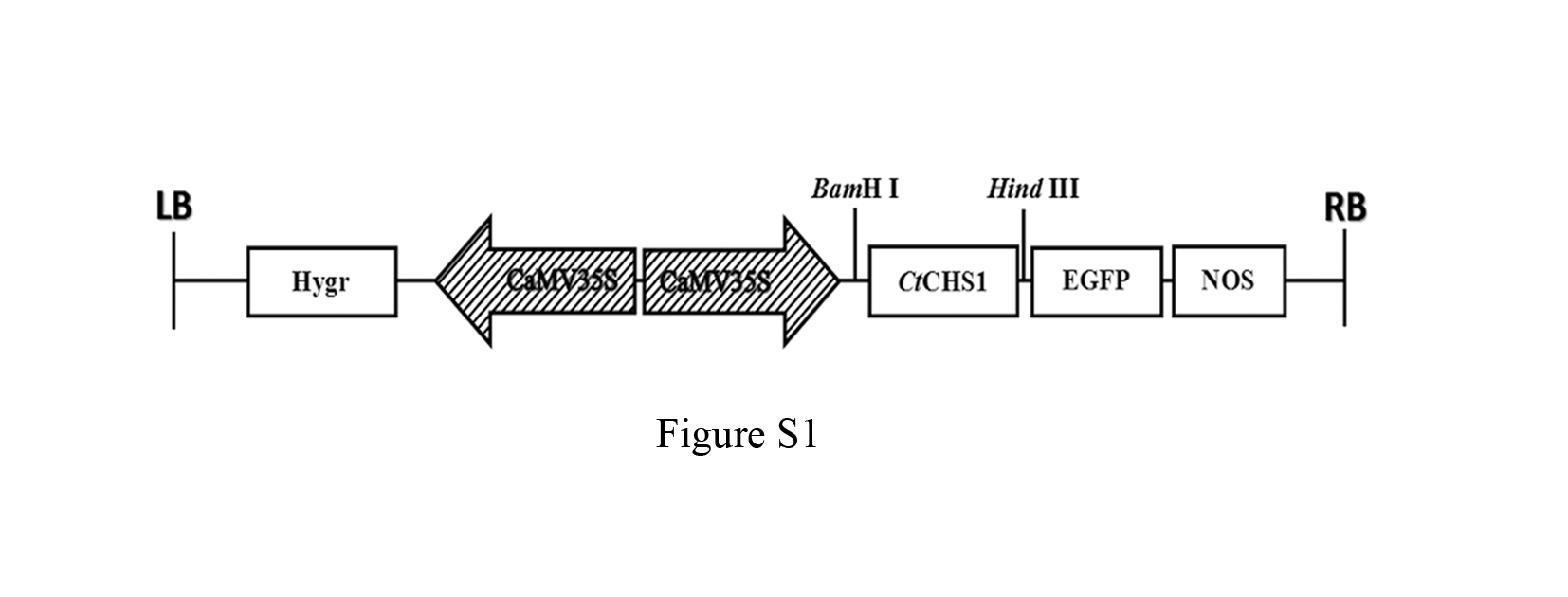

Supplement: FIGURE S1 — Plasmid map. [file Image_1.TIF]

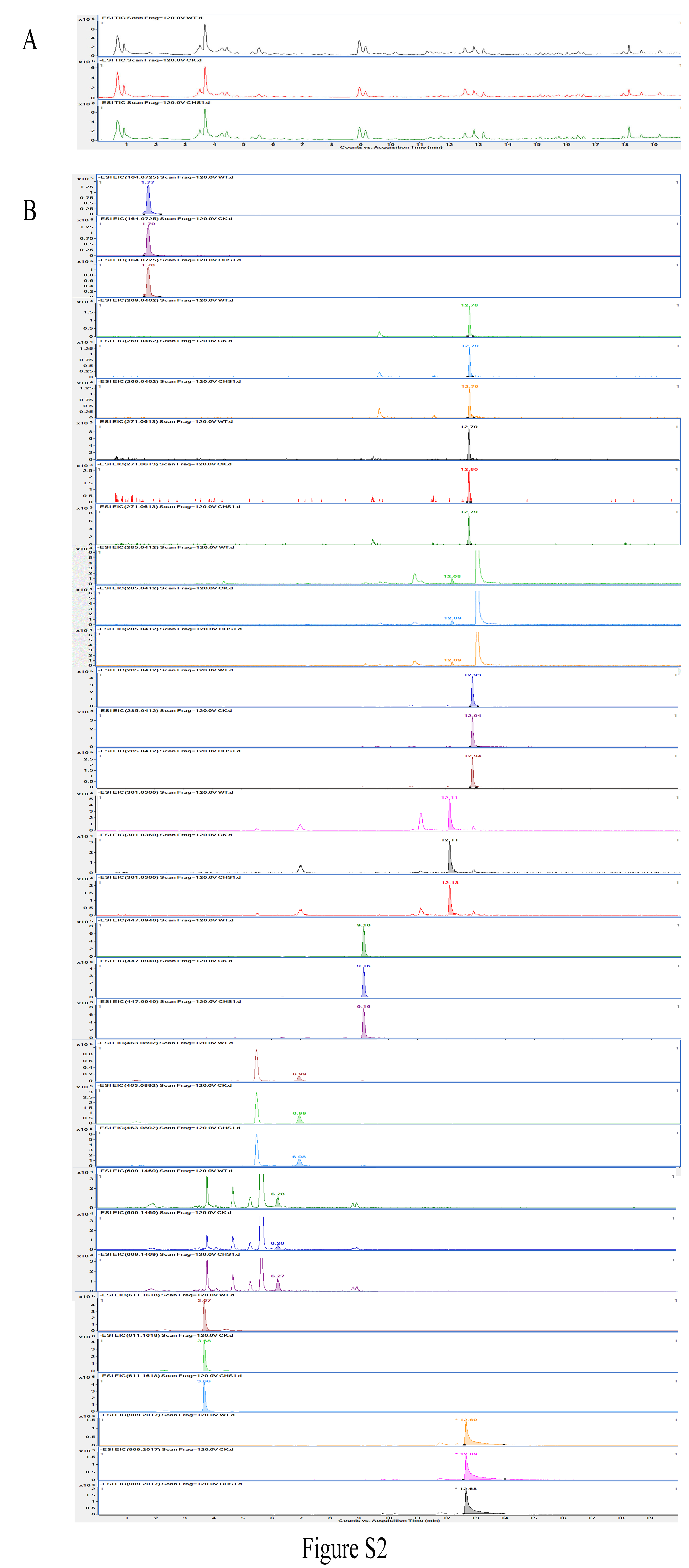

Supplement: FIGURE S2 — Representative total ion chromatograms (TICs) in ESI negative ion mode based on UPLC-Q-TOFMS from WT, CK and OVX in safflower. (B) EICs of each 11 metabolites from WT, CK and OVX in safflower. [file Image_2.TIF]
